# Entrapment of a volatile lipophilic aroma compound (d-limonene) in spray dried water-washed oil bodies naturally derived from sunflower seeds (*Helianthus annus*)^☆^

**DOI:** 10.1016/j.foodres.2013.08.024

**Published:** 2013-11

**Authors:** Ian D. Fisk, Robert Linforth, Gil Trophardy, David Gray

**Affiliations:** a Division of Food Sciences, University of Nottingham, Sutton Bonington Campus, Sutton Bonington, Loughborough, Leicestershire, LE12 5RD, United Kingdom; b Firmenich SA, Avenue du Mail 15, 1205 Geneva, Switzerland

**Keywords:** Flavour, Aroma, Water-washed oil bodies, Encapsulation, Aroma encapsulation, Spray drying

## Abstract

Oil bodies are natural emulsions that can be extracted from oil seeds and have previously been shown to be stable after spray drying. The aim of the study was to evaluate for the first time if spray dried water-washed oil bodies are an effective carrier for volatile lipophilic actives (the flavour compound d-limonene was used as an example aroma compound). Water-washed oil bodies were blended with maltodextrin and d-limonene and spray dried using a Buchi B-191 laboratory spray dryer. Lipid and d-limonene retention was 89–93% and 24–27%. Samples were compared to processed emulsions containing sunflower oil and d-limonene and stabilised by either lecithin or Capsul. Lecithin and Capsul processed emulsions had a lipid and d-limonene retention of 82–89%, 7.7–9.1% and 48–50%, 55–59% respectively indicating that water-washed oil bodies could retain the most lipids and Capsul could retain the most d-limonene. This indicates that whilst additional emulsifiers may be required for future applications of water-washed oil bodies as carriers of lipophilic actives, oil bodies are excellent agents for lipid encapsulation.

## Highlights

• Spray dried water-washed oil bodies were prepared with d-limonene and maltodextrin
• Water-washed oil bodies were compared to processed emulsions
• Water-washed oil bodies were most effective at retaining lipid during spray drying

## 1 Introduction

Oilseeds store energy in the form of triglycerides for periods of dormancy in preparation for germination and the early stages of seed development. Triglyceride is stored in discrete subcellular organelles that are formed as a result of the synthesis of neutral lipids within the bilayer of the cellular endoplasmic reticulum; as lipid accumulates it forms droplets of insoluble material that swell, distending the endoplasmic reticulum membrane, and at a critical diameter, separate from the endoplasmic reticulum by vesiculation forming independent organelles termed oil bodies. These organelles are natural lipid emulsion droplets structurally stabilised by their residual phospholipid monolayer (60% phosphatidylcholine, 20% phosphatidylinositol) (Furse et al., 2013) and a series of highly amphiphilic proteins (78% oleosin, caleosins and steroleosins) (Furse et al., 2013).

Oil bodies have previously been exemplified as vehicles for post extractive entrapment of lipophilic compounds (Fisk, Linforth, Taylor, & Gray, 2011), and further studies have focused on the ability of oil bodies to extract functional components, in particular oil bodies from species containing bioactive compounds have been studied (Adams et al., 2012; Iwanaga et al., 2007; Nantiyakul et al., 2012) and have shown that oil bodies can not only accumulate functional lipophilic compounds (internally or through passive external association), but can also be used as extraction vehicles for tocopherols (Fisk, White, Carvalho, & Gray, 2006), isoflavanoids (Fisk & Gray, 2011), tocotrienols (White, Fisk, & Gray, 2006) and oryanols (Nantiyakul et al., 2012). Furthermore the digestive fate of oil bodies has recently been investigated by Hoad and Gallier who proposed that the oil body chemical composition and structural design contribute to a retardation of digestive pepsinolysis and pancreatic lipolysis (Gallier & Singh, 2012; Gallier, Tate, & Singh, 2012; Hoad et al., 2008).

Compounds once entrapped within or on the structure of oil bodies will partition on a vapour pressure basis from the hydrophobic internal core to the surrounding air. This partitioning will be dependent on a number of interacting factors (Fernandez-Vazquez et al., 2013). The two main controlling parameters are the rate of mass transfer from the matrix (controlled by diffusion kinetics and/or mechanical driving forces) (Fisk, Boyer, & Linforth, 2012; Yang et al., 2012; Yu et al., 2012); and the physicochemical and thermodynamic properties of the aroma molecule and matrix itself (vapour pressure, diffusivity, solubility, partition coefficient and activity coefficients) (Buttery, Guadagni, & Ling, 1973; Carey, Asquith, Linforth, & Taylor, 2002; Doyen, Carey, Linforth, Marin, & Taylor, 2001), the latter being crudely summarised by the aroma compounds partition coefficient (Eq. (1)).

(1) $$K_{i}=\frac{C_{v}}{C_{l}}$$

Partition coefficient (*K*) of compound *i*. Where the concentration of *i* in the vapour phase (v) and in the liquid phase (l) is shown by *C*_v_ and *C*_l_ respectively.

To enhance the functionality of a carrier for a volatile lipophilic compound, two factors can therefore be modified. The diffusion or mixing kinetics of the active from the core to the external phase, and the equilibrium concentration difference between the internal and external phase. Given that we are not concerned with mixing kinetics in this study and that the equilibrium partition coefficient cannot be altered without changing the fundamental chemical composition of the carrier, the greatest opportunity to enhance entrapment stability lies in the control of diffusion kinetics. This can be seen commercially in the wide use of glassy state hydrocolloids to stabilise volatile or reactive compounds through melt-extrusion or spray drying processes (Madene, Jacquot, Scher, & Desobry, 2006).

In their native state oil bodies are stored in high sugar, low moisture content (5–10%) conditions (Murphy, 2001), and destabilise during hydration and germination. It is therefore likely that oil bodies will be stable if stored in comparable conditions after extraction. It has previously been shown for omega-3 enriched *Helianthus annus* and *Echium plantagineum* oil bodies that the rate of progression of oxidative rancidity is relatively slow in extracted oil bodies (Fisk, White, Lad, & Gray, 2008; Gray, Payne, McClements, Decker, & Lad, 2010) and can be further slowed by drying in a glassy matrix (Gray, 2012), although it is dependent on the choice of drying technology and presence of metal ions (Kapchie, Yao, Hauck, Wang, & Murphy, 2013). Furthermore Matsakidou, Biliaderis, and Kiosseoglou (2013) has recently shown that composite films can be formed through air drying with a sodium caseinate carrier and several other authors have shown that extrinsic proteins, functional hydrocolloids or thermal pre-treatment can modify the stability of extracted oil bodies (Chen, McClements, Gray, & Decker, 2012; Karkani, Nenadis, Nikiforidis, & Kiosseoglou, 2013; Nikiforidis, Kiosseoglou, & Scholten, 2013; Wu et al., 2011).

This study builds on the concept that oil bodies are inherently stable unit structures and tests the hypothesis that if treated carefully oil bodies can be extracted and dried to form stable structures ex-vivo with the potential to act as carriers for lipophilic actives. 4-Isopropenyl-1-methylcyclohexene (d-limonene) was used as a model compound and processed emulsions stabilised by a surface active modified food starch (Capsul) or a surface active lipid (lecithin) were used for comparison (as a commercial benchmark and to represent a phospholipid stabilised system respectively).

## 2 Materials and methods

### 2.1 Materials

Capsul (oxylsuccinated starch) was sourced from National Starch, Yorkshire, UK. Morex (Maltodextrin 10-19 DE) and d-limonene was sourced from Firmenich SA, Geneva, Switzerland. Dehulled sunflower seeds (China) were less than 20 months old and were sourced from Lembas Ltd, UK. All other chemicals were purchased from Sigma-Aldrich, Gillingham, UK.

Germination tests (Zarbakhsh, Prakash, & Shetty, 1999) were performed on 25 seeds (*n* = 5) on hydrated filter paper by the ‘between paper’ method (International_Seed_Testing_Association, 1985). Dehulled sunflower seeds were shown to have acceptable germinability (60%).

### 2.2 Oil body isolation

Water-washed or urea-washed oil bodies from dehulled sunflower seeds (100 g batches) were extracted (grinding medium 0.5 L, 10 mM sodium phosphate buffer pH 7.5, 0.6 M sucrose) and purified (5 mL per 25 mL, 10 mM sodium phosphate buffer, pH 7.5) by methods detailed previously (Fisk et al., 2011, 2008), originally based on the method of Tzen, Peng, Cheng, Chen, and Chiu (1997). Urea-washed oil bodies were used to evaluate the impact of spray drying on the basic oil body size distribution (Fig. 2) and water-washed oil bodies were used for all other results, water-washed oil bodies were chosen as they represent the commercially viable fraction that may be used for future applications and at laboratory scale had extraction yields of > 10% had lipid concentrations of > 80% and protein concentrations of < 10%, SDS-PAGE protein characterisation was conducted and has confirmed the presence of oleosin and caleosin proteins (data not shown).

### 2.3 Size distribution

Particle size distribution was measured using a Malvern Mastersizer S (Malvern Instrument, Malvern., England) equipped with a small sample dispersion unit and a 300RF lens. The volume particle size distribution was calculated using the Malvern Mastersizer S polydisperse analysis model (3NAD presentation code, 1.095 real refractive index, 0 imaginary refractive index, 1.33 dispersant refractive index, 15% obscuration), all samples were dispersed in 10 mM sodium phosphate buffer (pH 7.5) for analysis.

Samples were dispersed by sonication for 60 s prior to pipetting dropwise into the small sample dispersion unit and allowed to equilibrate for 60 s prior to measurement.

### 2.4 Spray drying and emulsion formulation

Oil (oil body or sunflower oil), water and carrier (Capsul or lecithin) were homogenised (Ultraturax T25; Janke-Kunkel, IKA Labortechnik) for two minutes at either 8000 rpm or 24,000 rpm (low or high homogenisation power) allowed to dissolve (2 hr) with d-limonene (Firmenich, Geneva, Switzerland) in a thermally controlled (35 °C) air-tight water bath and stirred with a dissolver disk (Vorsicht RW20) at 240 rpm. Morex was added and stirred for a further 30 min, samples were further homogenised (Ultraturax T25; Janke-Kunkel, IKA Labortechnik) for one minute at either 8000 rpm or 24,000 rpm (low or high homogenisation power) prior to spray drying.

All samples had a 30% ± 5% solids content composed of 11% d-limonene, the remaining 89% was composed of either 62.6% Morex, 22% dry weight of water-washed oil bodies neutral lipid for the oil body sample, 4.4% non-lipid oil body material (calculated by calculating the neutral lipid content at 22% and adding Morex to achieve a solids content of 30%); 53% Morex, 22% sunflower oil and 14% lecithin for the lecithin sample and 53% Morex, 22% sunflower oil and 14% Capsul for the Capsul sample. Sample experimental design was chosen to directly compare samples at equal oil concentrations (22%), lecithin (phospholipid) was added as oil bodies contain a surface coating of phospholipid, and Capsul was chosen as a commercial standard for stabilising lipophilic volatiles. Morex is not surface active and serves to enhance the glassy state transition temperature of the spray dried product, which enhances stability during dry storage (Soukoulis, Behboudi-Jobbehdar, Yonekura, Parmenter, & Fisk, 2013).

Spray-drying was performed using a Buchi B-191 Laboratoriums-Technik (Flawil, Switzerland) test spray dryer. The spray dryer was operated at 180 °C and 85 °C inlet and outlet temperatures respectively; aspirator at 90% and sample pump at 10%. Drying air was filtered nitrogen at 650 mL min^−1 ^ and filter pressure drop was maintained below 30 mBar.

Moisture content of spray dried products was calculated by the Karl–Fisher method (Firmenich SA, Geneva).

Samples were isolated and for short term storage (< 24 hr) were stored in air tight glass vials in desiccators containing lithium chloride (Behboudi-Jobbehdar, Soukoulis, Yonekura, & Fisk, 2013) for longer term storage (> 24 hr) samples were stored in double sealed air tight containers at 5 °C. All aroma analyses were carried out within 14 days of sample preparation.

### 2.5 Static headspace analysis by APcI-MS

**Fig. 1:**
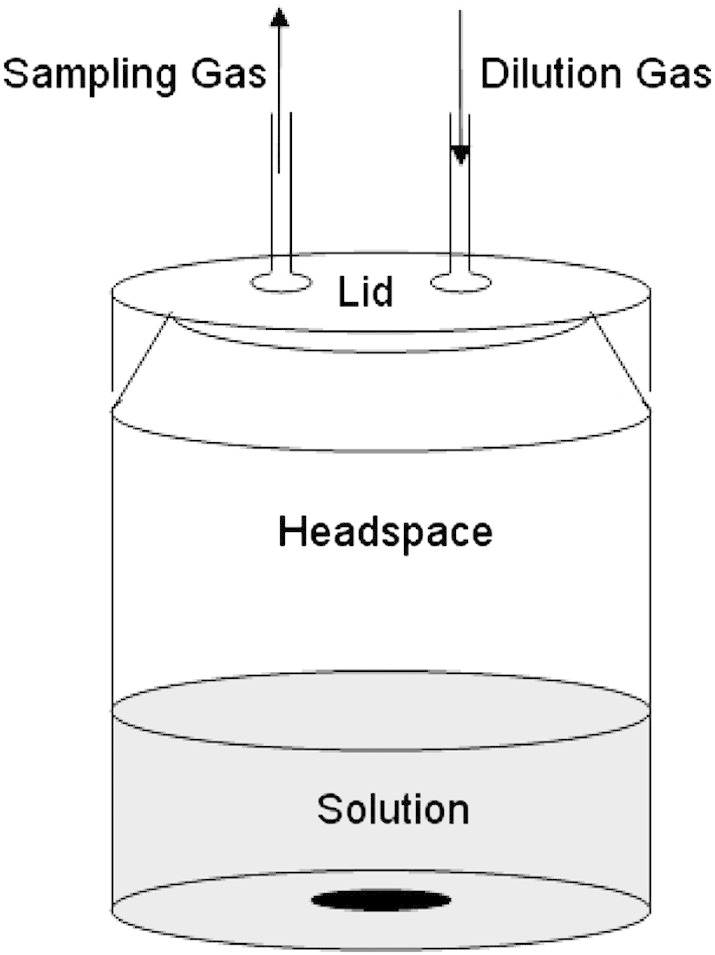
Dynamic headspace dilution apparatus.

Static headspace analysis was carried out as per Fisk et al. (2011), previously described by Linforth (1999), in brief, samples (10 mL) were placed in a capped Schott Bottle (volume 123 mL) with dissolution solvent and allowed to equilibrate for 2 hr (Fig. 1), then subjected to a direct injection mass spectrometry (APcI-MS, MS-Nose™, Micromass-LCZ, Micromass, Altrincham, UK) (flow rate 10 mL/min). Cone voltage and ion monitored was 15 V and 137 *m*/*z*, respectively (Fernandez-Vazquez et al., 2013).

Relative headspace intensity (RHI) for a volatile compound is calculated as per Doyen et al. (2001)

(2) $$\mathrm{RHI}\%=100\times \frac{C_{v\left(E\right)}}{C_{v\left(w\right)}}$$

Where RHI% = relative headspace intensity; *C*_v(E)_ = concentration of the volatile in the vapour phase above an emulsion sample and *C*_(w)_ = concentration of the volatile in the vapour phase above a buffer only sample.

d-Limonene concentration above the spray dried powders was calculated using the static headspace approach as detailed above, but the sampling gas flow was diluted with nitrogen at a flow rate of 3 L min^− 1^ in a 2 L dilution chamber. Absolute headspace concentration was calculated by direct injection of d-limonene standards in hexane (10 ppbv–10 ppmv) into the MS detector.

### 2.6 Dynamic headspace analysis by APcI-MS

Dynamic headspace analysis was performed according to the method previously described (Fisk et al., 2011), originally proposed by Doyen et al. (2001). Samples (100 mL) were allowed to equilibrate for 4 hr in Schott bottles (123 mL), and the headspace measured for volatile concentration during dynamic headspace dilution with Nitrogen gas (70 mL min^− 1^) through the dilution port (Fig. 1) and simultaneous analysis via the sampling gas port by APcI-MS as described previously (sampling flow rate 70 mL min^− 1^). Headspace dilution was continued for 10 min and headspace intensity was measured relative to the initial headspace intensity and expressed as percentage, hereinafter it is referred to as % normalised headspace intensity (%nHI). Samples were stirred throughout the measurement period.

### 2.7 d-Limonene quantification by standard addition

d-limonene content of spray dried powders was calculated by headspace standard addition using gas-chromatography-mass-spectroscopy (HSS-GC-MS) (Agilent model G1290B, G2613A, G1530A, G2578A). Samples (10 mg) were diluted in water (1 mL) sealed in 20 mL headspace vials with Teflon septa and shaken. Dilution water contained a serial dilution of d-limonene, such that the standard addition curve can be extrapolated to calculate the absolute concentration of d-limonene in the sample (*n* = 3, *R*^2^ < 0.999).

HSS Model G1290B parameters were 45 °C oven, 70 °C valve, 100 °C transfer line, 20 min equilibrium time, 0.05 min fill time, 1 min injection time. Oven temperature ramped from 60 °C to 240 °C at 40 °C min^− 1^, 50:1 split ratio, sampling flow rate 75 mL min^− 1^, Agilent column 19091S-433 HP-5MS, 0.25 mm, 30 m. G2578A settings were 150 °C MS quad, 250 °C source. d-Limonene was identified at 3.13 min using 93.10, 121.10 and 107.10 as identifier ions.

### 2.8 Statistical analysis

All preparations were carried out in triplicate, statistical differences (*P* > 0.05) were calculated using analysis of variance-Tukey's or LSD (Zhang, Linforth, & Fisk, 2012) using XLSTAT 2011, Addinsoft, UK).

## 3 Results and discussion

**Fig. 2:**
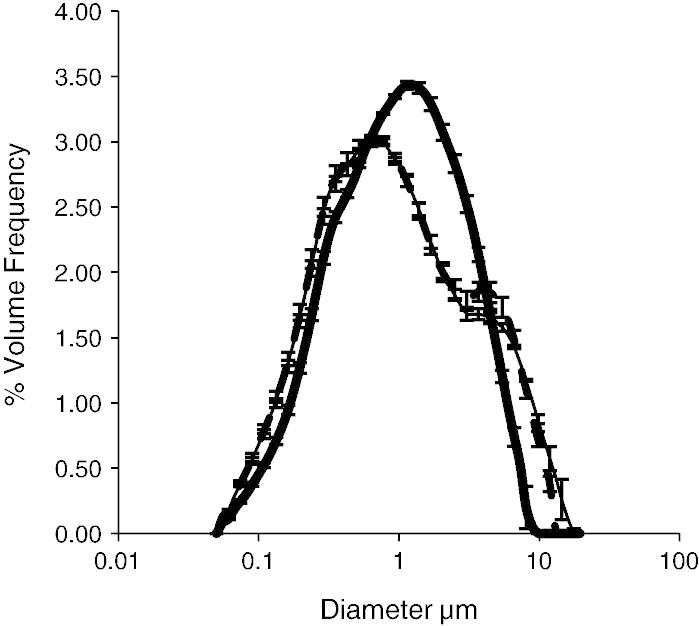
Volume based size distribution of urea-washed sunflower oil bodies (UW) before (thick black line) and after spray drying and rehydration (1 hour (dotted line) and 24 hours (thin black line) after rehydration).

To evaluate the stability of the basic unit structure of oil bodies, oil bodies were prepared, purified by urea washing and spray dried. Urea-washed oil bodies had a tight size distribution (~ 1–2 μm) and were stable to the spray drying process (Fig. 2). Oil bodies were prepared at 10% solids and initial trials showed that spray dried urea-washed sunflower oil bodies could produce a powder that was not greasy to touch and when rehydrated produced a stable suspension of oil bodies. Although after spray drying and rehydration some aggregation of the oil bodies within the powder did occur, this is shown in Fig. 2 where a small shoulder can be observed on the volume based size distribution plot at ~ 4 μm. To further investigate the validity of this result the dispersed sample was held for a further 23 hours and measured again using the same protocol; over the holding time there was no oiling out or phase separation and the fraction of 4 μm particles was reduced over the storage period (*P* < 0.05). This illustrates that the basic unit structure of oil bodies (isolated though urea-washing) are stable to spray drying and appear to be physically intact after rehydration.

As urea-washed oil bodies prepared at low concentration would not be commercially viable for the food industry, a higher concentration preparation of water-washed oil bodies (30%) was prepared in triplicate and used for subsequent evaluation. Water-washed oil bodies were subsequently dried at this higher feed concentration using a maltodextrin carrier (Morex 10–19 DE).

The higher concentration stock solution was chosen to maximise throughput and enhance aroma retention. It has previously been shown by Reineccius that aroma retention in a spray dried product can be enhanced with higher in-feed solids content (Reineccuis, 1989, 2004). The maltodextrin carrier (Morex 10–19 DE) was chosen for its intermediate DE (dextrose equivalent) value, its ability to form a glassy matrix on spry drying and current use in the food industry as a spray dry carrier. To identify if Morex impacted oil body stability, samples of Morex (20% *w*/*w*) in a water-washed oil body suspension (7% *w*/*w*) were sonicated twice for two minute pulses to test the effect of the additive on the stability of water-washed oil bodies. The samples containing Morex had a significantly fewer aggregates than the sample without. This confirmed that Morex was not detrimental to the physical stability of water-washed oil bodies, and may actually stabilise the suspension, which could be due to the increase in viscosity of the continuous phase.

Water-washed oil bodies were therefore mixed with maltodextrin and d-limonene and dried at 30% solids content by spray drying. This product was compared to two processed emulsions that were formed using sunflower oil, d-limonene and Morex, with either lecithin or Capsul (a modified starch) as an additional surfactant, d-limonene is a lipophilic cyclic terpene and is expected to partition into the oil phase of an emulsion system and thereby be stabilised.

Lecithin is a common phospholipid based, food grade emulsifier that is used in a wide range of food application, it was chosen to maximise comparability as oil bodies are partially stabilised by phospholipid. Capsul is an octenyl succinylated starch which has been chemically modified to increase aroma retention on spray drying (Porzio & Popplewell, 2002). The commercial effectiveness of Capsul is attributed to the fact that it rapidly enters the glassy state during drying and therefore subsequently confers physical and chemical stabilities to entrapped aroma compounds. Capsul was chosen as a commercial benchmark to compare against spray dried water-washed oil bodies.

To evaluate the homogenisation process and to ensure oil body stability was not compromised, two levels of homogenisation were used, high and low. Low homogenisation level was low shear mixing that should not damage the oil body integrity. High homogenisation level was high shear mixing that may damage the water-washed oil bodies, but was chosen to distribute, more evenly, the d-limonene in the in-feed stock suspension.

All products were spray dried successfully and formed dry, white-yellow powders. The Capsul powders were white, powdery, free flowing and felt crunchy when compressed. Lecithin powders were yellow-white in colour (author’s personal observations), tended to clump in aggregates and felt slightly oily; however, the high homogenisation lecithin sample clumped less and felt less oily. The oil body powders were white, free flowing and appeared denser than the Capsul samples (author’s personal observations). The variation in the products apparent density was probably due to variation in lipid content between the samples and the variation in perceived oiliness was probably due to the location of lipid in the spray dried particles, indicating that the Capsul stabilised samples had the lowest oil content and that the lecithin had the greatest free surface lipid. The variation in lipid content was confirmed by solvent extraction (Table 1).

All products were produced on a Buchi B-191 spray drier. The B-191 has a relatively small drying chamber (cylinder) with a high surface area to volume ratio. This is expected to impact spray dryer yields and although the B-191 will produce a reproducible spray dried powder, yields (Eq. (3)) will be lower than commercially and the thermal profile will be different to many industrial practices.

(3) $$\%\mathrm{Yield}=100\times \frac{W_{\mathrm{product}}}{W_{\mathrm{raw}\_\mathrm{material}}}$$

Percentage total yield (%yield), where: *W*_product_ and *W*_raw_material_ are weight of product and raw material respectively.

The yield (Eq. (3)) of lipid varied between the samples (Table 1). This was principally due to adhesion of the wet or oily product on the wall of the drying chamber, or loss through non-collection of small particles. The highest total product yield for all six products was that of the high homogenisation water-washed oil body preparation (24% dwb), and was significantly higher than that of the other five samples (12–18%) (Table 1). This is presumed to be due to several factors; the lecithin product is believed to have a higher amount of free lipid on the surface of the powder making it more adhesive and harder to transport to the cyclone, and the Capsul product was a very fine powder which may have increased its losses via the waste air (Table 2). To compensate for variation in total yield, hereinafter the samples shall be compared on a % retention basis (Table 2), this calculation removes the inefficiencies of the spray dryer as it calculates on a dry weight basis per gram of product and raw material and not on a total yield basis (Soottitantawat, Yoshii, Furuta, Ohkawara, & Linko, 2003). The absolute concentration of d-limonene and lipid is shown in Table 1 and the % retention of both lipid and d-limonene is shown in Table 2.

Water-washed oil bodies combined with Morex was the most effective carrier for spray drying lipid. The % lipid retention was 89–93% compared to a lipid retention of 48–50% for Capsul and 82–89% for lecithin. This shows a marked benefit of using water-washed oil bodies as a method of drying and storing lipid as a dry powder, further supporting the hypothesis that oil bodies are viable storage structures for lipid ex-vivo (Gray, 2012). Additional benefits include the antioxidant protection offered by Morex (Reineccuis, 2004) as free reducing groups in the maltodextrin will act as antioxidants to stabilise the entrapped lipid.

The most effective carrier for d-limonene was Capsul and water-washed oil bodies were shown to be significantly better carriers for d-limonene than lecithin (Table 1). The d-limonene retention differential between Capsul and oil body systems can be explained by the quantities of surface active material present and the physical state of the matrix. Morex has no emulsification properties (Reineccuis, 2004) and the emulsifiers present in the oil body system are actively involved in stabilising the lipid fraction, whilst the ability of the natural emulsifiers to retain lipid is high, in the natural oil body system there is no redundancy in surface activity potential, therefore there is no remaining surface active material to stabilise the d-limonene. It is proposed that partially defatted oil bodies or additional oleosin or phospholipid be added to enhance the d-limonene retention further in future studies or commercial applications of these findings. The lecithin system was least effective in stabilising d-limonene and this could be partially explained by the depression of its glass state transition temperature by phospholipids (lecithin) and subsequent increase in diffusivity within the matrix.

To evaluate the physical stability of the entrapped oil bodies over 6 month’s shelf life, spray dried water-washed oil bodies were stored at 5 °C for 6 months. Powders were re-suspended in buffered water at 30% (wwb) and the particle size of the resulting oil emulsion measured (Table 3) after equilibrium. In all samples the low homogenisation preparation produced bimodal distributions (data not shown) with peaks at 1 μm and 10 μm. The original numerical mean particle diameter was 1–2 μm and after storage the numerical mean particle diameter for the water-washed oil bodies ranged from 2.02 μm to 3.18 μm, indicating some aggregation or coalescence but in general a high level of oil body stability. It is presumed that the 10 μm shoulder represents either aggregates or coalesced emulsion droplets. For the alternative carrier matrices the high homogenisation samples consistently showed a bimodal distribution but the 10 μm peak was significantly smaller than the low homogenisation samples. In conclusion, the three high homogenisation samples produced emulsion droplets of comparable mean size to the original sample (Table 3) and all spray dried samples did not lose their physical stability over a 6 month storage period.

It is generally accepted that small emulsion droplets in the in-feed solution have higher aroma retention (Reineccuis, 2004) so stability during production and storage is key to the functionality of a carrier for volatile actives. Soottitantawat et al. (2003) suggested that a reduction in particle size diameter would enhance retention and reduce loss of d-limonene; this mechanism is proposed to be due to shearing of the large droplets by the spray dryer atomiser, their rupture allowing a selective loss of volatile through volatilisation.

The headspace concentration of d-limonene was evaluated above the dry carrier and above the rehydrated powder (0.015% lipid). To facilitate direct comparison between the data the dry powder headspace concentration and rehydrate headspace intensity are shown in Table 4 standardised on a lipid and d-limonene basis. Capsul stabilised samples after high homogenisation had a significantly lower d-limonene headspace concentration when in a dry system compared to the water-washed oil body preparation (*P* < 0.05) although there was no difference in the low homogenisation samples of Capsul and water-washed oil bodies. Lecithin had the lowest dry powder headspace concentration which can be explained by the hydrophobicity of lecithin and resultant increase in concentration of non-polar material in the lecithin sample. The lower dry powder headspace concentration of Capsul further indicates the effective physical encapsulation of d-limonene in the glassy state.

After suspension at 0.015% lipid content, there were no significant differences in rehydrate headspace intensity between Capsul and water-washed oil body products (compared between homogenisation levels at *P* < 0.05) or between lecithin and water-washed oil body products, although there was a significant difference between the lecithin and the Capsul products (ANOVA-Tukey's), there was no impact of homogenisation level (*P* < 0.05). It should be noted that differences in product structure can impact aroma delivery during preparation and hydration (Fisk, Kettle, Hofmeister, Virdie, & Silanes Kenny, 2012; Yu et al., 2012) as well during equilibrium and this should be considered prior to future applications in complex foods.

**Fig. 3:**
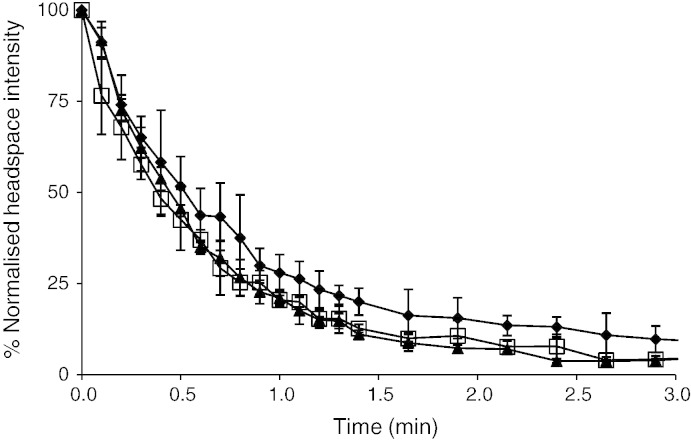
Normalised time-course profiles for dynamic headspace dilution measurements of d-limonene in an aqueous suspension of spray dried samples stabilised by water-washed oil bodies (diamond), lecithin (triangle) and Capsul (cross), all samples were prepared with low homogenisation and at 0.015% lipid sample, experiment was run for 10 min for all samples, but data are only shown to 3.0 min for clarity.

Further to the static headspace measurements of d-limonene, the spray dried rehydrated water-washed oil bodies were assessed by a dynamic headspace dilution method. This was carried out on the powder after re-suspension to more closely represent the ability of oil body systems to release aroma compounds during consumption. The results for low homogenisation are shown in Fig. 3. Statistical analysis was performed (ANOVA, *P* < 0.05) and showed that there is no statistically significant differences between the data sets after 2.5 minutes. Although it can be noted that in all cases the samples subjected to low shear mixing had higher %nHI than those subjected to high shear mixing. It can also be noted that in both cases the %nHI of the oil body suspension was higher in the early phases of headspace dilution than the lecithin sample. This corroborates our earlier work oil body suspensions (Fisk et al., 2011), which showed that water-washed oil bodies had a higher normalised headspace intensity than phospholipid stabilised emulsions, although as this effect is lipid content dependent the low lipid levels have reduced the total measurable differences.

## 4 Conclusion

In conclusion, water-washed oil bodies were successfully spray dried using maltodextrin as a carrier, lipid retention was 89–93%, which indicates that the water-washed oil bodies are stable during spray drying and that an oil body-maltodextrin structure is a good approach for stabilising lipid. 4-isopropenyl-1-methylcyclohexene (d-limonene) was incorporated into the spray drier feed and spray drier retention was 24–27%, indicating that additional emulsifier may be required for future applications. Spray dried water-washed oil bodies were compared directly with spray dried sunflower oil emulsions stabilised with modified starch (Capsul); Capsul gave a significantly lower lipid retention of 48–50% but higher d-limonene retention of 55–59%.

## Figures and Tables

**Table 1:** Composition, extraction yield^a^ and d-limonene headspace concentration of spray dried water-washed oil bodies, and lecithin and Capsul stabilised processed emulsions.

	Oil body		Lecithin		Capsul	
	Low	High	Low	High	Low	High
Moisture (%)	3.86 ± 0.04	2.76 ± 0.03	3.45 ± 0.04	1.86 ± 0.05	3.69 ± 0.05	3.94 ± 0.01
Limonene (%)	2.68 ± 0.1	2.95 ± 0.04	0.85 ± 0.01	1.00 ± 0.02	6.00 ± 0.05	6.54 ± 0.2
Lipid (%)	20.5 ± 1	19.6 ± 2	29.6 ± 0.9	32.0 ± 1	10.9 ± 0.6	11.0 ± 1
Yield (%)	15.8	23.8	12.2	17.5	15.4	15.8

a % yield values shown in Table 1 represent total spray dried yield (dry weight of spray dried product/dry weight of raw material × 100).

**Table 2:** % Lipid and d-limonene retention^a^ of spray dried water-washed oil bodies, and lecithin and Capsul stabilised processed emulsions.

	Oil body		Lecithin		Capsul	
	Low	High	Low	High	Low	High
Lipid (%)	93 ± 1.0	89 ± 2.0	82 ± 0.9	89 ± 1.0	50 ± 1.0	48 ± 1.0
Limonene (%)	24 ± 0.1	27 ± 0.0	7.7 ± 0.0	9.1 ± 0.0	55 ± 0.1	59 ± 0.2

a Lipid and d-limonene retention shown in Table 2 is calculated as the % analyte retention per unit solids when comparing the raw ingredient to the final product, expressed on a percentage basis (100 × analyte concentration in spray dried product, dwb/analyte concentration in raw material product, dwb).

**Table 3:** Particle size of re-suspended spray dried powders (water-washed oil bodies, and lecithin and Capsul stabilised processed emulsions) after storage, (5 °C, 6 months) 30% solid content (numerical mean diameter, *D*(*v*,0.5)).

	Oil body		Lecithin		Capsul	
	Low	High	Low	High	Low	High
*D* (*v*,0.5)	3.2 μm	2 μm	2.9 μm	2.2 μm	3.4 μm	1.9 μm

**Table 4:** Static headspace d-limonene concentrations above dry and wet spray dried samples (water-washed oil bodies, and lecithin and Capsul stabilised processed emulsions) standardised on a d-limonene and lipid basis.

	Oil Body		Lecithin		Capsul	
	Low	High	Low	High	Low	High
Dry headspace^a^	20.3 ± 4	27.0 ± 5	6.51 ± 0.7	7.92 ± 1	17.6 ± 5	18.1 ± 4
Wet headspace^b^	83.9 ± 27	67.7 ± 12	58.0 ± 9	80.9 ± 8	121 ± 49	79.5 ± 23

a d-limonene static headspace concentration above dry powder (0.1 g) in 2 L container headspace measured by APcI-MS with internal standards.

b d-limonene static headspace intensity above sample (0.015% Lipid, w/w) re-suspended in 250 mL distilled water measured by APcI-MS.
